# Development of an indirect ELISA for the serodiagnosis of canine infection by *Onchocerca* *lupi*

**DOI:** 10.1038/s41598-024-53759-w

**Published:** 2024-02-09

**Authors:** Maria Stefania Latrofa, Viviane Noll Louzada-Flores, Carla Maia, Maureen A. Kelly, Guilherme G. Verocai, Cinzia Cantacessi, Domenico Otranto

**Affiliations:** 1 Department of Veterinary Medicine, University of Bari, Valenzano, 70010 Bari, Italy; 2 Global Health and Tropical Medicine, Associate Laboratory in Translation and Innovation Towards Global Health, Instituto de Higiene e Medicina Tropical, Universidade Nova de Lisboa, Rua de Junqueira 100, Lisboa, Portugal; 3 Department of Veterinary Pathobiology, School of Veterinary Medicine and Biomedical Sciences, Texas A&M University, College Station, TX 77843 USA; 4 Department of Veterinary Medicine, University of Cambridge, Cambridge, UK; 5 Department of Veterinary Clinical Sciences, City University of Hong Kong, Kowloon Tong, Kowloon, Hong Kong

**Keywords:** Infectious diseases, Proteomics

## Abstract

*Onchocerca* *lupi* is a zoonotic filarioid parasite of dogs and cats with widespread distribution. A specific non-invasive diagnostic assay for the detection of *O.* *lupi* infections remains unavailable. This study aimed to assess the accuracy, specificity, and sensitivity of an ELISA test designed using nine peptides from two *O.* *lupi* proteins. Sera (n = 54) collected from *O.* *lupi* infected dogs from endemic areas (Portugal and USA), alongside sera from dogs positive for *Dirofilaria* *immitis*, *D.* *repens*, *Cercopithifilaria* *bainae*, and *Acanthocheilonema* *reconditum* (n = 53) from a non-endemic area for *O.* *lupi*, as well as from helminth-free dogs (n = 60), were tested. The checkerboard titration method was applied for the optimization of peptide concentrations and conjugate anti-dog dilutions. Sensitivity, specificity, and optimal cut-off values were calculated using ROC curve analysis. All peptides reacted against sera of *O.* *lupi*, with no correlation between optic density (OD) values and microfilariae (mfs) loads. Sensitivity and specificity values ranging from 85.45 to 100%, and 88.89% to 100%, respectively, were recorded for all peptides examined, with 100% specificity and sensitivity observed for peptides 40_3, 40_5, 130_3, 120_3 and 40_1, 130_5, respectively. The maximum cut-off value was observed for peptides 40_5 (0.765) and 40_3 (0.708). Testing of sera from dogs positive for other filarioids resulted in lower OD values (up to 1.565) for peptides 40_3 and 40_5 when compared with *O.* *lupi* (up to 2.929). The availability of this assay will be of value in epidemiological studies of canine *O.* *lupi* infection in both endemic and non-endemic areas, and in assessing the risk for zoonotic transmission.

## Introduction

Over the past decade, *Onchocerca* *lupi* (Spirurida, Onchocercidae) has attracted growing interest from the scientific community across continents^1^. From original taxonomic description in a Caucasian wolf^2^, this filarioid nematode has been widely reported as a causative agent of ocular infection in domestic dogs and cats, as well as in wild carnivores (wolves, coyotes), particularly in Europe and North America^3–6^. In animals, *O.* *lupi* microfilariae (mfs) are found in the cutaneous tissues^7,8^, whilst adult worms reside in the ocular connective tissues (i.e., eyelids, conjunctiva, and sclera) and, although infections are often asymptomatic, clinical signs ranging from acute or chronic ocular disease (i.e., periorbital swellings, photophobia or blindness) may be observed^1,5,8^.

Notably, important gaps in knowledge of the fundamental biology of this parasite still remain, in particular regarding its arthropod vector. DNA of *O.* *lupi* was detected in the blackfly species *Simulium* *tribulatum*^9^ and *Simulium* *griseum*^5^, as well as in other blood feeding arthropods, e.g., mosquitoes or biting midges (*Culicoides* spp.)^10,11^.

In the early 2010s, a case of human infection by *O.* *lupi* was diagnosed in Turkey^12^. This report was subsequently followed by other reports of human onchocerciasis due to *O.* *lupi* in both Europe^12–16^ and the USA^17–21^, thus highlighting the urgent need for specific diagnostic tools to better understand the epidemiology of this zoonotic nematode.

In animal hosts, diagnosis of *O.* *lupi* infection relies on the identification of adult parasites in ocular nodules in symptomatic cases^7^, or on ultrasound examination and computed tomography in asymptomatic animals^22^. Regardless of the imaging techniques, ultimately, diagnosis is achieved by morphological and molecular analyses of subcutaneous mfs in skin biopsies^7,8,23^. However, this diagnostic approach is invasive, time-consuming, and may lead to false-negative results, since mfs detection is highly dependent on their anatomical location, density, prepatent period and/or previous microfilaricidal treatments, as well as on operator skills^8^. The performance of serological enzyme-linked immunosorbent assay (ELISA) kits developed for the detection of antibodies against *Onchocerca* *gibsoni* (i.e., Og4C3)^24^ and *Dirofilaria* *immitis* (DiroCHEK^®^, SNAP^®^ Heartworm and SNAP^®^ 4Dx^®^ Plus)^25^, have been evaluated for serodiagnosis of *O.* *lupi* infection or to assess any cross-reactivity by testing sera from dogs with confirmed onchocerciasis. Similarly, the sensitivity and specificity of a western blot assay against *O.* *lupi* paramyosin^26^ has been evaluated, as well as the immunogenic properties of six reactive peptides from *O.* *lupi* Paramyosin (*Ol*-PARA) and Major Antigen (*Ol*-MJA)^27^. However, the reactivity of these peptides against sera of dogs positive for *O.* *lupi* infection is yet to be demonstrated.

This study aimed to assess the accuracy, specificity, and sensitivity of an indirect ELISA targeting a total of nine peptides, including the six linear epitopes previously characterized from *Ol*-PARA and *Ol*-MJA and three additional peptides from *Ol*-MJA.

## Results

### Identification of novel peptides and indirect ELISA optimization

**Figure 1:**
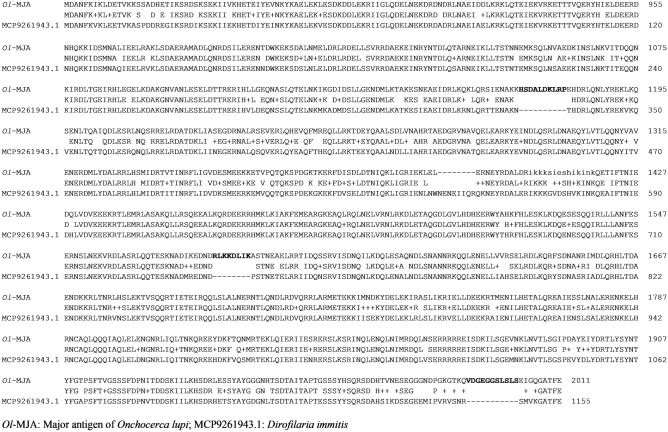
Alignment of amino acid sequences of Major Antigen of *Onchocerca* *lupi* and of spindle-and centrosome-associated protein of *Dirofilaria* *immitis*. Peptides 40_1, 40_3 and 40_5 are indicated in bold.

**Figure 2:**
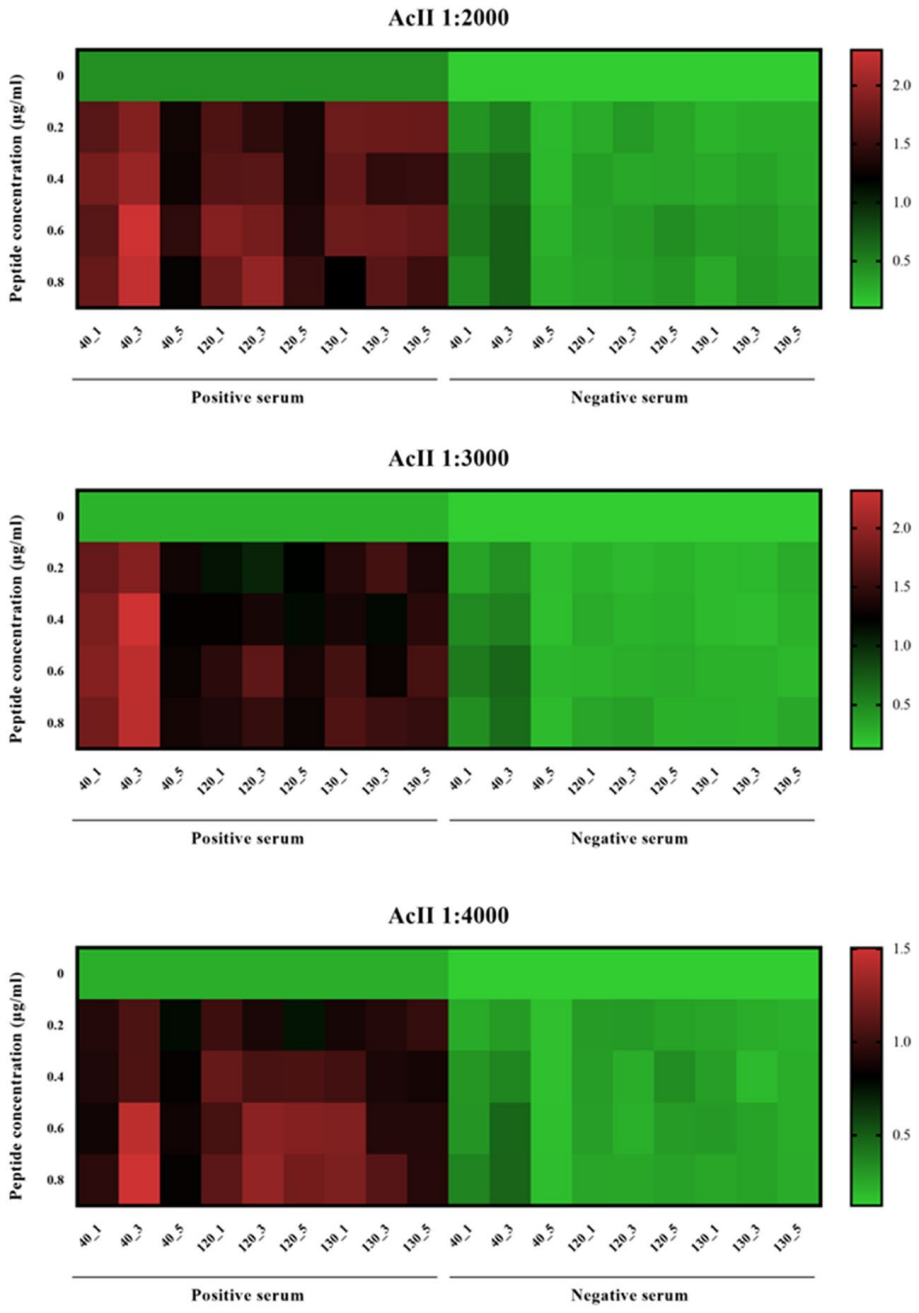
Heatmap from positive and negative serum samples for *Onchocerca* *lupi* tested against different concentrations of peptides and anti-dog conjugate dilutions at 1:2000, 1:3000 and 1:4000.

**Figure 3:**
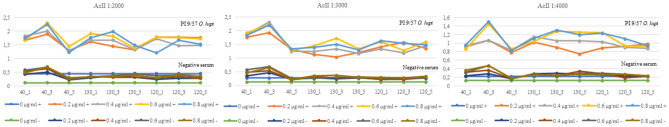
Checkerboard titration of each peptide and anti-dog dilution. Raw data obtained from positive and negative reference sera are indicated by dots. + :  Positive serum; −:  Negative serum.

BLASTp analysis of the amino acid sequence of *Ol*-MJA displayed the highest identity (83.08%) with GenBank MCP9261943.1, a spindle-and centromere-associated protein from *D.* *immitis*. Sequence alignment revealed a three peptide-insertion in *Ol*-MJA (aa 8 to 11) (Fig. 1), hereafter referred to as 40_1: HSDALDKLRP; 40_3: RLKKDLIK; 40_5: VDGEGGSLSLS. All peptides were confirmed to be immunoreactive against the reference serum P1*Ol* 9/57 by indirect ELISA (Table 1). Checkerboard titration revealed an optimum peptide concentration of 0.2 μg/ml and an optimal dilution of anti-dog conjugate at 1:3000. The optimum P/N ratio was observed for all peptides examined, except for 130_1, 130_3 and 130_5 (Table 2). OD values up to 0.431 were observed as background binding for 0 μg/ml peptide concentration with horseradish peroxidase (HRP) anti-dog conjugate dilution at 1:2000 (Figs. 2, 3).

**Table 1:** Serum samples from *Onchocerca* *lupi* infected dogs identified according to adult, microfilaridermia detection (mfs loads) and identification and country of collection.

ID samples	Country	Positivity	Mfs load	Proteins								
				OD Major antigen peptides						OD Paramyosin peptides		
				130_1	130_3	130_5	40_1	40_3	40_5	120_1	120_3	120_5
OL-4	USA	Clinical suspicion, serum only	–	0.698	0.759	0.581	0.839	1.098	0.802	2.048	0.775	0.633
OL-5	USA	Clinical suspicion, serum only	–	0.929	0.978	0.784	0.785	1.428	0.896	1.128	0.881	0.681
OL-7	USA	Clinical suspicion, serum only	–	1.854	1.824	1.569	1.298	2.001	1.389	1.624	1.754	0.816
OL-8	USA	Adult: qPCR	–	1.926	1.944	1.584	0.833	1.950	1.520	1.484	1.300	1.220
OL-9	USA	Clinical suspicion, serum only	–	0.995	1.233	1.001	0.984	1.392	1.538	1.228	0.975	0.981
OL-14	USA	Adult: qPCR	–	1.184	1.151	0.987	1.405	1.359	1.217	1.388	1.169	1.291
OL-17	USA	Clinical suspicion, serum only	–	0.933	1.081	0.923	0.918	1.173	1.313	1.316	1.022	0.944
OL-19	USA	Adult: qPCR	–	0.549	0.656	0.485	0.846	0.795	0.660	0.764	0.566	0.691
OL-22	USA	Clinical suspicion, serum only	–	0.713	0.765	0.632	0.736	0.674	0.934	1.123	0.762	0.441
OL-24	USA	Adult: qPCR	–	1.105	1.266	0.904	1.258	1.461	1.412	1.475	0.881	0.805
OL-28	USA	Adult: qPCR	–	0.980	0.961	1.451	1.131	1.276	1.016	0.984	0.886	0.879
OL-30	USA	Conjunctival tissue: qPCR	–	1.142	1.129	1.003	1.191	1.251	1.089	1.406	1.340	1.218
OL-32	USA	Conjunctival tissue: qPCR	–	0.585	0.766	0.420	0.707	0.835	0.587	0.691	0.573	0.588
OL-33	USA	Conjunctival tissue: qPCR	–	0.546	0.717	0.564	1.290	1.531	1.333	1.039	0.806	0.752
OL-34	USA	Conjunctival tissue: qPCR	–	0.375	0.438	0.444	0.780	0.767	0.928	0.422	0.348	0.402
OL-35	USA	Adult: qPCR	–	0.794	0.938	0.786	0.995	1.544	1.097	1.358	1.045	1.168
OL-36	USA	Clinical suspicion, serum only	–	0.603	0.610	0.566	0.558	0.720	0.713	0.729	0.515	0.493
NINA	Portugal	Skin: mfs, qPCR	–	0.947	0.905	0.645	0.927	1.378	1.130	1.786	1.730	1.625
P0 *Ol* 9/57	Portugal	Skin: mfs, qPCR	35	2.044	1.891	1.224	1.492	2.464	2.026	2.366	1.728	1.892
P0 *Ol* 1/32	Portugal	Skin: mfs, qPCR	23	0.924	1.073	0.796	0.992	1.220	1.099	1.464	1.036	0.659
P0 *Ol* 3/4	Portugal	Skin: mfs, qPCR	2	0.936	0.456	0.433	0.707	0.835	0.587	0.416	0.309	0.343
P0 *Ol* 6/58	Portugal	Skin: mfs, qPCR	1	0.936	0.731	1.059	1.039	1.079	1.237	1.626	1.200	1.045
P0 *Ol* 11/53	Portugal	Skin: mfs, qPCR	1	1.915	1.466	1.686	1.307	1.477	1.623	2.020	1.871	1.819
P1 *Ol* 1/32	Portugal	Skin: mfs, qPCR	4	0.924	1.106	0.952	0.992	1.220	1.099	0.858	0.890	1.017
P1 *Ol* 10/63	Portugal	Skin: mfs, qPCR	1	2.205	2.157	1.867	1.855	2.659	2.884	2.384	2.257	2.486
P1 *Ol* 7/42	Portugal	Skin: mfs, qPCR	4	0.519	0.533	0.518	0.781	0.955	0.871	0.858	0.890	1.017
P1 *Ol* 9/57	Portugal	Skin: mfs, qPCR	18	2.314	2.054	1.067	1.815	2.366	1.768	0.910	0.810	1.572
P1 *Ol* 6/58	Portugal	Skin: mfs, qPCR	1	1.740	1.798	1.695	1.803	2.286	2.123	1.905	1.776	1.943
P2 *Ol* 6/58	Portugal	Skin: mfs, qPCR	1	2.643	2.962	3.270	2.254	2.727	2.757	2.334	1.634	1.998
P2 *Ol* 11/53	Portugal	Skin: mfs, qPCR	1	2.021	2.104	2.024	1.517	1.736	1.949	1.766	1.521	1.448
P2 *Ol* 1/32	Portugal	Skin: mfs, qPCR	2	1.079	1.384	1.021	0.859	1.171	1.314	1.078	0.839	0.847
P2 *Ol* 7/42	Portugal	Skin: mfs, qPCR	1	1.453	1.237	1.358	1.586	1.593	2.362	1.480	1.637	1.515
P2 *Ol* 9/57	Portugal	Skin: mfs, qPCR	1	1.952	1.565	1.536	1.518	1.539	2.238	2.259	1.709	1.311
P2 *Ol* 10/63	Portugal	Skin: mfs, qPCR	1	1.107	1.092	0.983	1.124	1.348	1.487	1.418	1.185	1.349
P3 *Ol* 1/32	Portugal	Skin: mfs, qPCR	7	0.953	0.952	0.866	0.657	0.651	1.158	1.249	1.084	0.829
P3 *Ol* 7/42	Portugal	Skin: mfs, qPCR	3	0.575	0.668	0.681	0.591	0.753	0.791	0.597	0.671	0.641
P3 *Ol* 11/53	Portugal	Skin: mfs, qPCR	2	1.181	1.355	1.219	0.948	1.294	1.140	0.784	1.066	1.180
P3 *Ol* 9/57	Portugal	Skin: mfs, qPCR	9	1.935	1.375	1.652	1.277	1.670	1.939	1.882	1.609	1.490
P4 *Ol* 1/32	Portugal	Skin: mfs, qPCR	5	1.318	1.601	1.336	1.392	0.928	1.288	1.209	0.952	1.125
P4 *Ol* 3/4	Portugal	Skin: mfs, qPCR	5	0.503	0.579	0.400	0.592	0.922	0.655	0.684	0.734	0.877
P4 *Ol* 5/55	Portugal	Skin: mfs, qPCR	8	2.425	1.424	1.446	1.469	1.512	1.592	1.786	1.619	1.719
P4 *Ol* 7/42	Portugal	Skin: mfs, qPCR	14	0.543	0.704	0.618	0.551	0.568	0.853	0.909	0.850	0.875
P4 *Ol* 9/57	Portugal	Skin: mfs, qPCR	29	1.454	1.255	1.304	1.499	1.567	1.646	1.730	2.014	1.624
P4 *Ol* 10/63	Portugal	Skin: mfs, qPCR	10	2.117	1.887	1.879	2.132	2.929	2.323	2.031	1.863	2.342
Lindo	UK^a^	Adult: qPCR	–	0.601	0.545	0.763	0.575	0.829	0.765	0.636	0.599	0.543
RUCA 15	Portugal	Skin: mfs, qPCR	–	1.497	1.186	1.255	1.593	2.111	2.507	1.418	1.253	1.393
Peluda 14	Portugal	Skin: mfs, qPCR	–	1.176	0.793	0.892	0.774	1.547	1.701	1.188	1.002	1.061
OLD 14	Portugal	Skin: mfs, qPCR	–	1.231	1.010	1.247	0.946	1.544	1.185	1.193	1.352	1.382
Labrador	Portugal	Skin: mfs, qPCR	–	1.775	1.678	1.803	1.725	2.087	2.555	1.629	1.490	1.510
CMD5	Portugal	Skin: qPCR	nd	0.935	1.049	0.855	0.482	0.559	0.497	0.372	0.474	0.405
CM D11	Portugal	Skin: qPCR	nd	1.034	1.516	1.354	0.685	0.708	0.787	0.922	0.947	0.721
CM D13	Portugal	Skin: qPCR	nd	1.151	0.952	1.394	1.216	0.895	2.556	1.865	1.765	2.072
CM D18	Portugal	Skin: qPCR	nd	1.013	1.059	1.324	0.707	1.265	1.161	1.033	0.754	0.892
CM D21	Portugal	Skin: qPCR	nd	1.156	1.030	1.726	1.882	2.530	2.042	1.689	1.278	2.502

The optical density (OD) value for each peptide examined is indicated.

qPCR: quantitative PCR.

^a^Animal imported from Portugal.

**Table 2:** OD450 ratio (P/N value) between *Onchocerca* *lupi* positive (P1*Ol* 9/57) and negative (helminth free dog) reference sera, according to anti-dog conjugate dilutions and peptide concentrations tested.

Peptide concentration	Peptides								
	40_1	40_3	40_5	130_1	130_3	130_5	120_1	120_3	120_5
Anti-dog conjugate dilution 1:2000									
0 μg/ml	4.2	4.2	4.2	4.2	4.2	4.2	4.2	4.2	4.2
0.2 μg/ml	4.0	3.8	6.1	5.7	3.9	4.3	7.5	6.5	6.5
0.4 μg/ml	3.4	3.2	5.9	4.8	5.5	4.2	6.0	4.5	5.4
0.6 μg/ml	3.0	3.4	5.7	5.7	5.1	3.1	4.8	4.6	5.4
0.8 μg/ml	3.6	3.3	4.3	5.5	5.7	3.8	4.1	4.2	4.3
Anti-dog conjugate dilution 1:3000									
0 μg/ml	2.1	2.1	2.1	2.1	2.1	2.1	2.1	2.1	2.1
0.2 μg/ml	5.0	4.5	6.3	4.2	4.2	4.6	6.5	6.7	4.4
0.4 μg/ml	3.9	4.3	6.3	4.1	5.1	4.1	6.0	5.6	5.3
0.6 μg/ml	3.4	3.3	5.7	5.6	6.0	4.4	5.8	4.7	6.6
0.8 μg/ml	4.0	3.4	6.1	4.1	4.1	4.6	5.8	5.8	4.7
Anti-dog conjugate dilution 1:4000									
0 μg/ml	1.8	1.8	1.8	1.8	1.8	1.8	1.8	1.8	1.8
0.2 μg/ml	3.9	3.7	4.8	3.6	3.1	2.8	3.5	4.1	4.6
0.4 μg/ml	3.0	3.0	5.1	4.1	4.6	3.1	3.7	4.9	3.9
0.6 μg/ml	2.8	3.1	5.1	3.8	5.7	4.4	4.2	3.5	4.1
0.8 μg/ml	2.6	3.2	4.8	4.4	5.2	4.5	4.9	4.2	4.1

The highest P/N values were observed for peptides 40 (up to 6.3 for 40_5) and 120 (up to 6.7 for 120_3). In addition, peptide 40_3 resulted in the highest OD values (i.e., up to 2.318) when testing P1*Ol* 9/57 for all anti-dog conjugate dilutions and peptide concentrations examined (Fig. 3). The minimum background noise was observed using the blocking reagent (Roche), which was confirmed as optimum for the indirect ELISA.

### Initial validation of peptides for diagnosis of *Onchocerca lupi* infection by indirect ELISA

When testing canine sera from dogs positive for *O.* *lupi*, the highest (3.270) and lowest (0.309) OD values were recorded for peptides 130_5 and 120_3, respectively (Table 1). No correlation was observed between OD values and skin mfs burden for any of the peptides (Table 1).

The optimal discrimination and the best predictive performance of the ELISA assay, determined by AUC values (ranging from 0.9603 for peptide 120_3 to 0.9959 for peptide 130_1), were confirmed by analysis of the ROC curves of positive sera for all peptides (Table 3). Sensitivity and specificity values ranging from 85.45% to 100%, and 88.89% to 100%, respectively, were recorded for all peptides examined. In particular, the highest specificity (100%) was observed for peptides 40_3, 40_5, 130_3 and 120_3, whilst peptides 40_1 and 130_5 returned the highest sensitivity (100%) (Table 3). The highest optimal cut-off value (0.765) was observed for peptide 40_5, followed by 40_3 (0.708); the lowest cut-off value was detected for peptides 40_1 (0.4) and 130_5 (0.42) (Table 3).

**Table 3:** Receiver operating characteristic (ROC) data for serum samples of dogs with confirmed or clinically suspected *Onchocerca* *lupi* infection.

Peptides	Optimal cut-off	Sensitivity (%)	Specificity (%)	PPV (%)	NPV (%)	AUC
Major antigen						
40_1	0.4	100	88.89	94.83	100	0.9838
40_3	0.708	92.59	100	100	87.1	0.9918
40_5	0.765	88.89	100	100	81.82	0.9842
130_1	0.503	96.3	96.3	96.43	100	0.9959
130_3	0.533	94.55	100	100	90	0.9933
130_5	0.42	100	88.89	94.83	100	0.9838
Paramyosin						
120_1	0.684	89.09	96.3	98	81.25	0.9704
120_3	0.671	85.45	100	100	77.14	0.9603
120_5	0.633	87.27	96.3	97.96	78.79	0.9727

PPV: positive predictive value; NPV: negative predictive value; AUC: area under the curve.

### Sensitivity and specificity of indirect ELISA testing using canine sera positive for other filarioid nematodes

**Figure 4:**
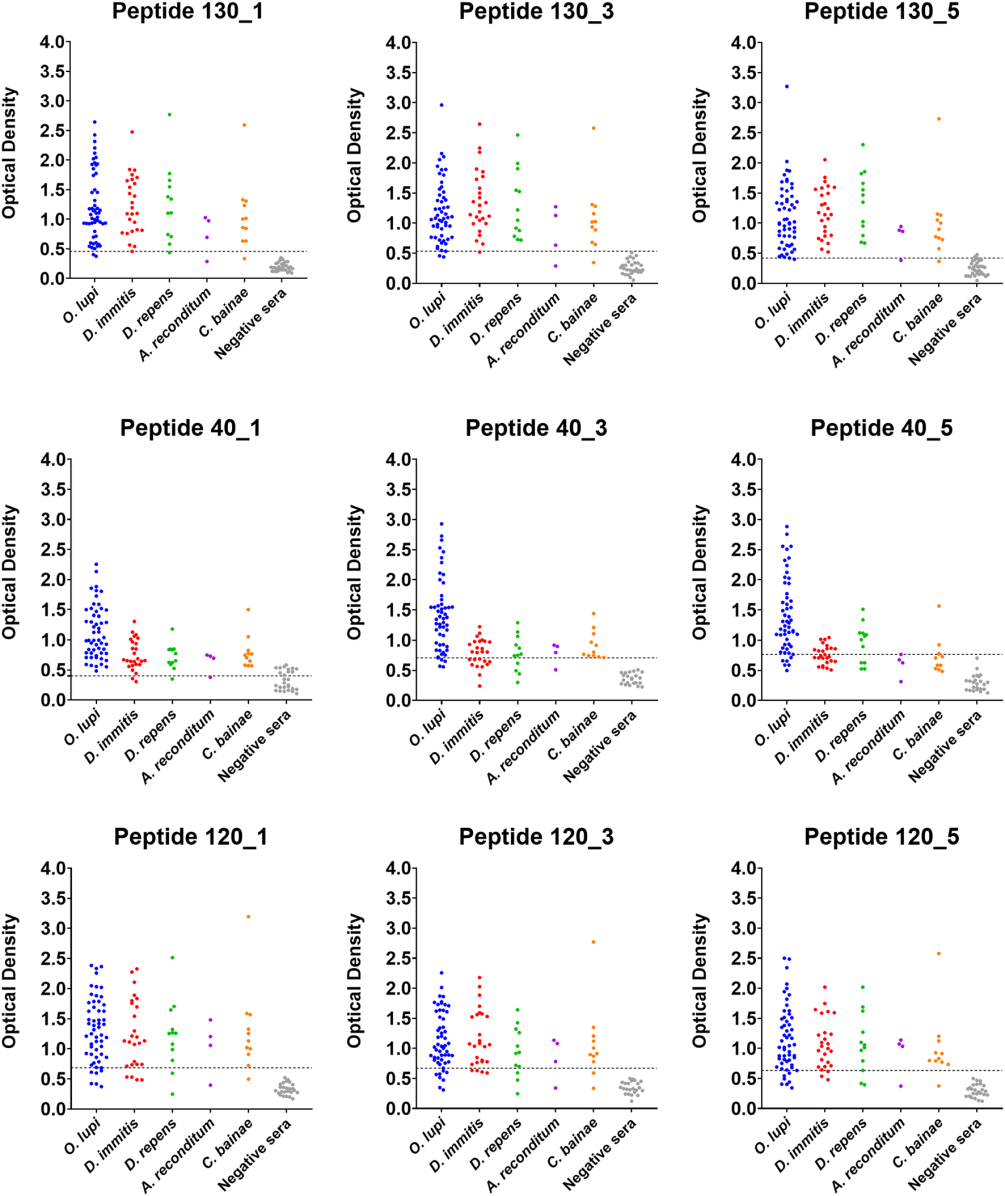
Optical density (OD) obtained for all peptides examined according to each pathogen and negative control sera.

ROC analysis returned the lowest AUC values for peptide 40_1 (0.8833) followed by peptide 120_3 (0.8611) against sera of dogs positive for *Acanthocheilonema* *reconditum*. The highest AUC values (= 1) were observed for peptide 130_1 against *D.* *immitis* and *D.* *repens*, and 130_3 and 130_5 against *D.* *repens* (Table 4). Overall, lower specificity values (ranging from 75 to 96.43%) were recorded for peptides 40_1, 40_3 and 40_5 against sera of dogs positive for other filarioid nematodes. Conversely, a specificity of 100% was observed for peptides 130 and 120 (Table 4). Lower OD values were observed for peptides 40_3 and 40_5 against *D.* *immitis*, *D.* *repens*, *A.* *reconditum* and *Cercopithifilaria* *bainae* (OD up to 1.565), when compared to those observed against *O.* *lupi* (OD up to 2.929) (Fig. 4). Overall, suboptimal OD cut-off values (up to 0.613 for 40_3, against *C.* *bainae*) were observed for peptides 40 when compared with those against *O.* *lupi* (up to 0.765 for 40_5) (Tables 3 and 4).

**Table 4:** ELISA parameters according to peptides of Major Antigen and Paramyosin proteins tested against serum samples of dogs infected by other common canine filarioid nematodes.

Peptides	Filarioid nematodes																							
	*Dirofilaria* *immitis*						*Dirofilaria* *repens*						*Cercopithifilaria* *bainae*						*Acanthocheilonema* *reconditum*					
	Cut-off	Se (%)	Sp (%)	PPV (%)	NPV (%)	AUC	Cut-off	Se (%)	Sp (%)	PPV (%)	NPV (%)	AUC	Cut-off	Se (%)	Sp (%)	PPV (%)	NPV (%)	AUC	Cut-off	Se (%)	Sp (%)	PPV (%)	NPV (%)	AUC
Major antigen																								
40_1	0.557	84.62	85.71	61.2	85.71	0.8846	0.591	83.33	88.89	76.92	92.31	0.9182	0.569	100	80.65	62.5	100	0.9323	0.696	75	92.59	60	96.15	0.8333
40_3	0.559	92.31	78.57	80	91.67	0.8997	0.493	91.67	77.78	64.70	95.45	0.9198	0.613	100	96.43	90.9	100	0.9893	0.575	90.91	96.3	90.9	96.30	0.9798
40_5	0.507	100	75	78.79	100	0.9066	0.523	100	82.14	70.59	100	0.9435	0.484	100	77.78	64.7	100	0.8889	0.625	75	81.84	37.5	95.65	0.787
130_1	0.456	100	100	100	100	1	0.579	100	100	100	100	1	0.335	100	92.59	84.61	100	0.9933	0.286	100	88.89	57.14	100	0.9722
130_3	0.654	96.15	100	100	96.55	0.9973	0.718	100	100	100	100	1	0.645	90.91	100	100	100	0.9798	0.633	75	100	100	96.43	0.9074
130_5	0.52	100	94.44	92.86	100	0.9968	0.668	100	100	100	100	1	0.575	90.91	96.3	90.9	96.3	0.9798	0.382	100	85.19	100	100	0.963
Paramyosin																								
120_1	0.711	84.62	96.43	95.65	87.1	0.9602	0.807	83.33	96.3	90.9	92.86	0.9167	0.723	90.91	96.15	90.9	96.15	0.972	1.056	75	100	100	100	0.9167
120_3	0.734	84.62	100	84.61	87.5	0.9815	0.697	75	100	100	90	0.8981	0.779	81.82	100	100	93.10	0.936	0.782	75	100	100	96.43	0.8611
120_5	0.535	96.15	89.29	89.28	96.15	0.9835	0.632	83.33	95.12	83.34	95.12	0.9451	0.733	90.91	100	100	96.43	.09697	1.036	75	100	100	96.43	0.9167

Se: sensitivity; Sp: specificity; PPV: positive predictive value; NPV: negative predictive value; AUC: area under curve.

## Discussion

In this study, we assessed the performance of an indirect ELISA based on primary antibody detection using specific peptides from two *O.* *lupi* proteins as antigens. We showed that all peptides are highly immunoreactive, also when tested against sera of dogs with low *O.* *lupi* microfilaridermia (i.e., OD = 2.643, mfs = 1). Our findings indicate that this non-invasive serological test may be applied to the detection of asymptomatic and/or amicrofilaremic infections, as well as of infections associated with aberrant sites of worm localisation^28–30^. Furthermore, the high positive predictive values (PPV) observed for some peptides (100% for 40_3, 40_5, 130_3 and 120_3) underscores the ability of this assay to discriminate between true vs. false positive results. Testing of dog sera with *O.* *lupi* infections revealed the high diagnostic accuracy of the indirect ELISA, as demonstrated by the high value of the AUC (> 0.9), as well as of specificity (100%) and sensitivity (from 85.45 to 94.55%) recorded for some of the peptides belonging to the Major Antigen (40_3, 40_5, 130_3) and Paramyosin (120_3) proteins. In addition, the robustness of this assay is also demonstrated by the high cut-off values recorded for peptide 40_3 and 40_5 (i.e., 0.708 and 0.765, respectively). However, the ELISA displayed a moderate cross-reactivity with lower specificity when peptides 40_3 and 40_5 were tested against canine sera from dogs infected by other filarioids (i.e., from: 75% for *D.* *immitis* to 96.43% for *C.* *bainae*), as well as lower overall cut-off threshold (up to 0.613 for *C.* *bainae*) and OD values (OD up to 1.565 for *C.* *bainae*) when compared with those for *O.* *lupi* (OD up to 2.929). The latter observation is of particular relevance, as it indicates that the ELISA with peptides 40_3 and 40_5 may support the diagnosis of canine *O.* *lupi* infection, also given the lower cut-off values for other filarioid nematodes (i.e., *D.* *immitis*, *D.* *repens*) that might be responsible for co-infections^5,31–33^ of dogs living in endemic areas, such as USA and Portugal^34,35^. Furthermore, these data may suggest that the ELISA may support screening of *D.* *immitis*-experimentally infected dogs^36^.

A limitation of this study is the unavailability of sera from dogs infected by other helminth species; nevertheless, the cross-reactivity between sera of dogs for which *O.* *lupi* infection is either suspected or confirmed and those of dogs infected with the most common filarioid species was assessed. In particular, moderate to high reactivity (sensitivity, 100%; specificity, 100%; OD ~ 2.5) was observed for some peptides against sera from animals positive for *D.* *immitis* and *D.* *repens* (i.e., peptides 130) and *C.* *bainae* and *A.* *reconditum* (i.e., peptides 120). These data contrast our previous finding obtained using microarray-based epitope mapping^27^ and highlight the limitations of this technology for high-throughput screening of sera^37^.

Overall, based on our data, peptides 40_3 and 40_5 yielded the best results for screening of canine *O.* *lupi* infection. Nevertheless, given that, thus far, no other ‘gold standard’ is available for diagnosis of canine onchocerciasis, we recommend that, until further validation can be carried out using additional independent assays, our ELISA test should be paired with microscopy-based and molecular detection tests, including conventional and real-time PCR^7,8,38,39^.

Moreover, although beyond the aim of our study, the reactivity of peptides 120 and 130 against infections by other filarioid species (*C.* *bainae* and *A.* *reconditum* and *D.* *immitis* and *D.* *repens*), deserves further investigation, as does the applicability of our assay to the diagnosis of feline infection by *O.* *lupi*. Indeed, cases of feline infections by this parasite are increasingly being reported (e.g., in Portugal, USA, and Romania), thus raising questions on the potential role of cats as reservoir of infection^40–42^. Furthermore, given that cases of *O.* *lupi* infections are being identified in animals from geographical areas where this parasite is considered non-endemic (e.g., UK) or of previously unknown endemicity (Israel)^43,44^, alongside cases of human infection^5,12^, the availability of a rapid, specific and sensitive tool for serodiagnosis of *O.* *lupi* infection is urgently needed, as it will assist the implementation of surveillance programmes aimed to investigate the geographic distribution and the epidemiology of this parasite. In turn, knowledge of *O.* *lupi* distribution will enhance current understanding of parasite epidemiology and fundamental biology, as well as risk of zoonotic transmission. Such efforts may also be aided by the determination of the *O.* *lupi* genome and/or transcriptome, and subsequent identification of additional epitopes for specific and accurate diagnosis of infection.

## Materials and methods

### Ethics statement

**Figure 5:**
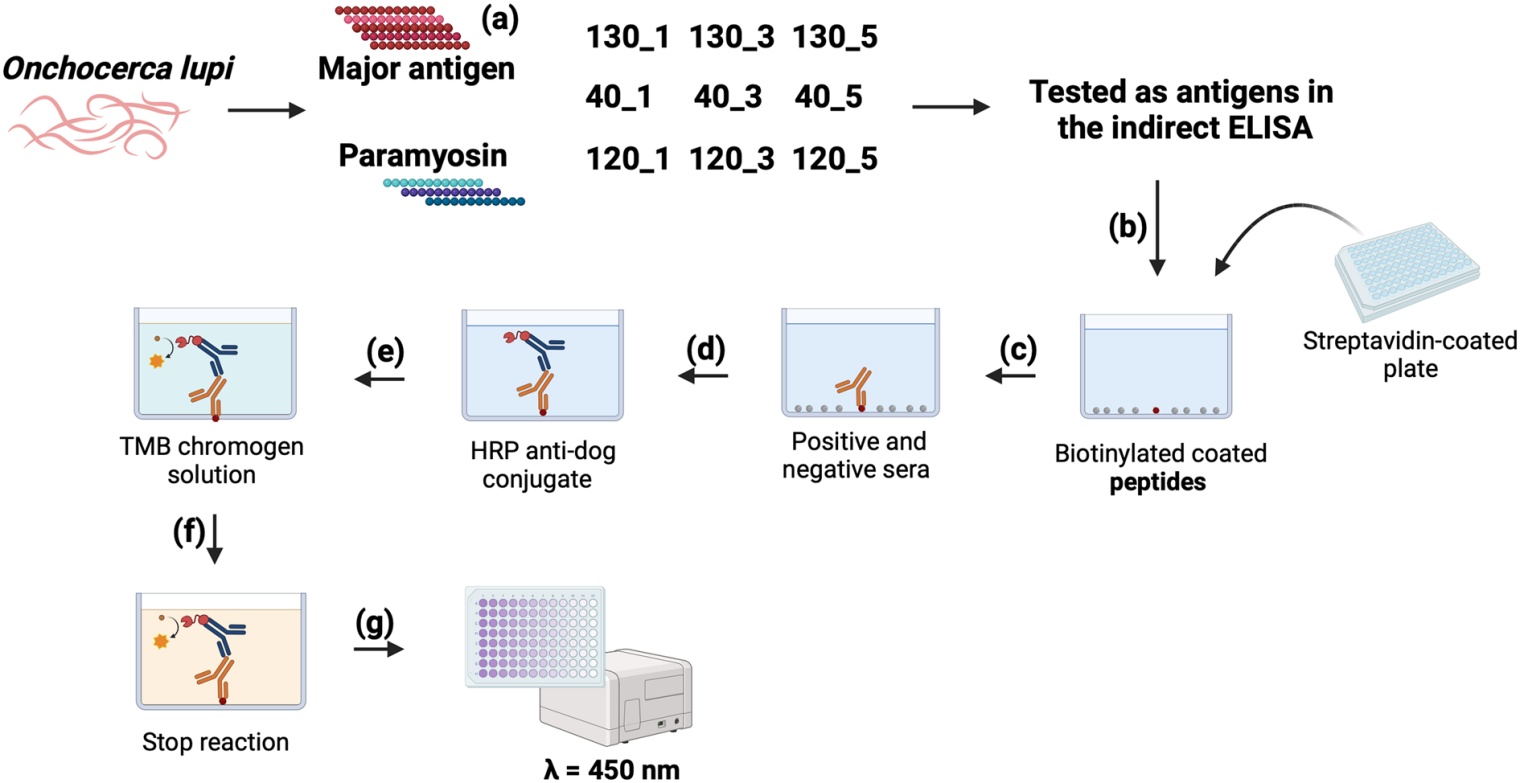
Schematic flowchart outlining the development of the indirect ELISA assay for serodiagnosis of *Onchocerca* *lupi*. Identification of new peptides from Major antigen protein (**a**). Coated peptides (antigen) onto wells of ELISA plate (**b**) interact with the first antibody from positive and negative canine serum samples (**c**). Adding the secondary antibody (conjugated antibody-HRP) (**d**). The reaction is developed by adding a substrate (**e**) which is cleaved by the conjugated enzyme and changes the reaction color after incubation (**f**). Results are read by ELISA plate reader (**G**). The figure was created with BioRender.com.

The study was conducted according to the Guidelines on Good Clinical Practices (The European Agency for the Evaluation of Medicinal Products, Veterinary Medicines and Information Technology Unit, VICH Topic GL9; https://www.emea.eu.int/pdfs/vet/vich/059598en.pdf). The procedures were approved by the ethical commission at the University of Èvora (identification number: AE02Fila2013), complying with Portuguese legislation for the protection of animals (Decree-Law no. 113/2013), by Texas A&M University’s Approval of Animal Use Protocol (IACUC 2022-0261 CA) and by the Ethics Committee of the Department of Veterinary Medicine of the University of Bari, Italy (Prot. Uniba 12/20). The methods were carried out in accordance with the regulations of the university and with the recommendations in the ARRIVE guidelines. A flowchart outlining the procedures leading to the development of the indirect ELISA assay described in this study is available from Fig. 5.

### Identification and synthesis of *Onchocerca lupi* linear peptides

The amino acid (aa) sequence of *Ol*-MJA protein^27^ was compared with those of Onchocercidae (taxid: 6296) and Nematoda (taxid: 6231) species, available from the NR protein database, using BLASTp (https://blast.ncbi.nlm.nih.gov/Blast.cgi?PAGE=Proteins)^45^.

The newly identified peptides, alongside those previously described from *Ol*-MJA and from *Ol*-PARA (^27^; *Ol*-MJA: 130_1: LQNDQLQSEIQRLR; 130_3: IGRIEKLELERNEY; 130_5: QREAIESSLNALE; *Ol*-PARA: 120_1: LEEARRRLE; 120_3: SRLQSEVEVLIVDL; 120_5: MQVDEEHKMF) were synthesized as biotinylated synthetic peptides with a (Gly)_4_ linker (Purity ≥ 95%, N-Terminal modification, Biotin) and purchased from GenScript Biotech (Rijswijk, Netherlands). All peptides were tested as antigens in the indirect ELISA.

### Assay standardization

The checkerboard titration method^46^ was used for optimization of the peptide concentration and conjugate anti-dog dilutions. Briefly, streptavidin-coated High-Capacity 96 well Plates (Thermo Fisher Scientific, Rockford, USA) were activated and rinsed three times with 200 μl PBS + 0.1% Tween-20 (PBS-T, washing buffer). Plates were incubated overnight at 4 °C with 100 μl of the selected biotinylated peptides diluted from 0.2 to 0.8 μg/ml in carbonate buffer, pH 9.6. The plates were subsequently rinsed three times with PBS-T under continuous shaking at 300 rpm at 35 °C for 30 min, thereby eliminating unbound peptides. Plates were blocked with 200 μl Blocking Reagent (Roche Diagnostics, Mannheim, Germany, GmBH) at 35 °C, 300 rpm for 30 min and washed three times with PBS-T 01%. Blocking with other reagents (i.e., PBS + 1% skim milk powder, buffer solution with gelatine) was also tested. Plates were dried by inversion on paper towel and 100 μl of positive and negative reference serum samples subjected to dilution in PBS-T at 1:40 and then incubated for 1 h at 35 °C and 300 rpm. Plates were washed four times with PBS-T and, once completely dried, incubated with 100 μl of HRP anti-dog conjugate, diluted at 1:2000, 1:3000 and 1:4000 (Invitrogen goat anti-canine IgG, Thermo Fisher Scientific, Waltham, USA), at 35 °C and 300 rpm for 1 h. After washing and drying, plates were incubated with 100 μl of TMB chromogen solution (Tetramethyl Benzidine, Sigma-Aldrich, St. Louis, Missouri, USA) for 10 min at room temperature. The colorimetric reaction was terminated with 50 μl stop solution (Invitrogen, Thermo Fisher Scientific, Vienna, Austria). The plate was then read using Absorbance 96 Plate reader Enzo (Byonoy, Hamburger, Germany) at a wavelength (λ) of 450 nm. The optimal conditions were selected based on the highest OD450 ratio between reference positive *O.* *lupi* (P1*Ol* 9/57) and negative serum samples (P/N value), testing all peptides with concentration ranging from 0 to 0.8 μg/ml. Background binding was assessed by testing peptides at concentrations ranging from 0 to 0.8 μg/ml with positive *O.* *lupi* and negative reference sera and with dilutions of HRP anti-dog conjugate at 1:2000, 1:3000 and 1:4000, respectively.

### ELISA validation using field canine sera

Sera from dogs with either suspected or confirmed *O.* *lupi* infection (n = 54)*,* available from previous studies conducted in endemic areas of Portugal (n = 37^31^) and the USA (n = 17^25^) were tested (Table 1), alongside sera (n = 53) from dogs living in a non-endemic area for *O.* *lupi* (Apulia and Sicily regions, Italy) that had previously tested positive for common filarioids of dogs (i.e., *D.* *immitis*, *D.* *repens*, *C.* *bainae* and *A.* *reconditum*) (Supplementary Table S1^32,38,47–49^). Sera from young dogs (n = 60) that had tested molecularly or serologically negative for helminth infections were also included as negative controls.

### Statistical analysis for determination of cut-off value and ELISA sensitivity and specificity

The diagnostic sensitivity (Se) and specificity (Sp) of the ELISA test, and the optimal cut-off, were calculated by plotting the receiver operating characteristic (ROC) curves (plots of sensitivity against [1 − specificity]). The area under the ROC curve (AUC) was estimated by non-parametric integration^50^ to measure diagnostic accuracy. ROC analyses were performed using Rstudio Version 1.6.0 with maximize metric method.

### Supplementary Information

Supplementary Table S1.

## Data Availability

All data analyzed during this study are included in this published article.
