# Supplementary material for: Development of an indirect ELISA for the serodiagnosis of canine infection by Onchocerca lupi
Source: Sci Rep. 2024 Feb 9;14:3348. doi: 10.1038/s41598-024-53759-w (PMC10858053; doi:10.1038/s41598-024-53759-w)
Supplement: Supplementary file 1 — Supplementary Table S1. [file 41598_2024_53759_MOESM1_ESM.docx]

**Supplementary Table S1.** Serum samples from *Dirofilaria repens, Cercopithifilaria bainae, Acanthocheilonema reconditum*, and *Dirofilaria immitis* tested by indirect ELISA.

| ***Dirofilaria repens*** | | | ***Cercopithifilaria bainae*** | | | ***Acanthocheilonema reconditum*** | | | ***Dirofilaria immitis*** | | | | | |
| --- | --- | --- | --- | --- | --- | --- | --- | --- | --- | --- | --- | --- | --- | --- |
| ID Sample | Origin | Positivity | ID Sample | Origin | Positivity | ID Sample | Origin | Positivity | ID Sample | Origin | Positivity | ID Sample | Positivity | Origin |
| 53 | Apulia | Blood, Skin: mfs, qPCR | 2_ 42610 | Apulia | Skin:  mfs, cPCR | 29_1_7155123 | Apulia | Blood: cPCR | 5 | Apulia | Blood, Skin: mfs, qPCR | 56 | Blood, Skin: mfs, qPCR | Apulia |
| 111 | Apulia | Blood, skin:  mfs, qPCR | 3 _ A4765 | Apulia | Skin: mfs, cPCR | 28_1_C2 | Apulia | Blood: cPCR | 15 | Apulia | Blood, Skin: mfs, qPCR | 58 | Blood, Skin: mfs, qPCR | Apulia |
| 119 | Apulia | Blood, skin:  mfs, qPCR | 4 _ A42636 | Apulia | Skin: mfs, cPCR | CE144 | Sicily | Blood: cPCR | 16 | Apulia | Blood, Skin: mfs, qPCR | 66 | Blood, Skin: mfs, qPCR | Apulia |
| 126 | Apulia | Blood, Skin:  mfs, qPCR | 5 _ A43100 | Apulia | Skin: mfs, cPCR | CE106 | Sicily | Blood: cPCR | 18 | Apulia | Blood, Skin: mfs, qPCR | 69 | Blood, Skin: mfs, qPCR | Apulia |
| 157 | Apulia | Blood, Skin:  mfs, qPCR | 7 _ A43100 | Apulia | Skin: mfs, cPCR |  |  |  | 19 | Apulia | Blood, Skin: mfs, qPCR | 71 | Blood, Skin: mfs, qPCR | Apulia |
| 174 | Apulia | Blood, Skin:  mfs, qPCR | 8 _ A42765 | Apulia | Skin: mfs, cPCR |  |  |  | 20 | Apulia | Blood, Skin: mfs, qPCR | 72 | Blood, Skin: mfs, qPCR | Apulia |
| 207 | Apulia | Blood, Skin:  mfs, qPCR | 9 _ A42813 | Apulia | Skin: mfs, cPCR |  |  |  | 27 | Apulia | Blood, Skin: mfs, qPCR | 74 | Blood, Skin: mfs, qPCR | Apulia |
| 226 | Apulia | Blood, Skin:  mfs, qPCR | 10 _ A31884 | Apulia | Skin: mfs, cPCR |  |  |  | 35 | Apulia | Blood, Skin: mfs, qPCR | 76 | Blood, Skin: mfs, qPCR | Apulia |
| 209 | Apulia | Blood, Skin:  mfs, qPCR | 11 _ A31642 | Apulia | Skin: mfs, cPCR |  |  |  | 44 | Apulia | Blood, Skin: mfs, qPCR | 82 | Blood, Skin: mfs, qPCR | Apulia |
| 6 | Apulia | Blood, Skin:  mfs, qPCR | 12 _ A43461 | Apulia | Skin: mfs, cPCR |  |  |  | 45 | Apulia | Blood, Skin: mfs, qPCR | 84 | Blood, Skin: mfs, qPCR | Apulia |
| 9 | Apulia | Blood, Skin:  mfs, qPCR | 13 _ A43462 | Apulia | Skin: mfs, cPCR |  |  |  | 46 | Apulia | Blood, Skin: mfs, qPCR | 86 | Blood, Skin: mfs, qPCR | Apulia |
| 49 | Apulia | Blood, Skin:  mfs, qPCR |  |  | Skin: mfs, cPCR |  |  |  | 47 | Apulia | Blood, Skin: mfs, qPCR | 90 | Blood, Skin: mfs, qPCR | Apulia |
|  |  |  |  |  |  |  |  |  | 54 | Apulia | Blood, Skin: mfs, qPCR | 95 | Blood, Skin: mfs, qPCR | Apulia |

cPCR: convetional PCR; qPCR: quantitative PCR.
